# Numerical Analysis of Degradation and Capacity Loss in Graphite Active Particles of Li-Ion Battery Anodes

**DOI:** 10.3390/ma15113979

**Published:** 2022-06-02

**Authors:** Jorge Marin-Montin, Mauricio Zurita-Gotor, Francisco Montero-Chacón

**Affiliations:** Materials and Sustainability Group, Department of Engineering, Universidad Loyola Andalucía, Avenida de las Universidades, s/n, 41704 Sevilla, Spain; jjmarin@uloyola.es (J.M.-M.); mzurita@uloyola.es (M.Z.-G.)

**Keywords:** Li-ion battery, active particles, graphite, capacity loss, modeling

## Abstract

It is well known that the performance and durability of lithium-ion batteries (LIBs) can be severely impaired by fracture events that originate in stresses due to Li ion diffusion in fast charge–discharge cycles. Existing models of battery damage overlook either the role of particle shape in stress concentration, the effect of material disorder and preexisting defects in crack initiation and propagation, or both. In this work we present a novel, three-dimensional, and coupled diffusive-mechanical numerical model that simultaneously accounts for all these phenomena by means of (i) a random particle generator and (ii) a stochastic description of material properties implemented within the lattice method framework. Our model displays the same complex fracture patterns that are found experimentally, including crack nucleation, growth, and branching. Interestingly, we show that irregularly shaped active particles can suffer mechanical damage up to 60% higher than that of otherwise equivalent spherical particles, while material defects can lead to damage increments of up to 110%. An evaluation of fracture effects in local Li-ion diffusivity shows that effective diffusion can be reduced up to 25% at the particle core due to lithiation, while it remains at ca. 5% below the undamaged value at the particle surface during delithiation. Using a simple estimate of capacity loss, we also show that the C-rate has a nonlinear effect on battery degradation, and the estimated capacity loss can surpass 10% at a 2C charging rate.

## 1. Introduction

The efficient management of energy and improvements in energy storage solutions are two of the main societal challenges today. Energy storage is needed to address imbalances between energy production and demand, as well as to provide solutions for delivery of off-grid electric power in portable consumer goods. Due to the inherently intermittent and unpredictable nature of energy produced from renewable sources and its increasing fraction in the total electricity production mix, there is an imperative need to improve energy storage technologies. In addition, the constant demand for ever-increasing energy hungry portable electronics and electric appliances and the need for cleaner mobility solutions such as the electric vehicle also require (and are made possible by) technological improvements in energy storage solutions.

Electrochemical batteries are portable devices that can deliver electrical power from stored chemical energy. They do so with high conversion efficiencies and without emissions [1,2,3,4]. Because of these features, electrochemical batteries are the most suitable solution today for portable energy applications; capacities range in size from small electronic devices to electric vehicles. In addition, electrochemical batteries are becoming practical solutions for energy storage in photovoltaic plants and have started to find use as large power supply equipment. In the context of the uncertainty of future demand, mainly due to the underestimation of the rate of technology change [5,6,7], the battery industry is currently pressed to increase battery scalability, performance, and durability, while simultaneously reducing production costs and environmental impacts [8,9,10,11,12,13].

Lithium-ion batteries play a prominent role in current electrochemical energy storage solutions, mainly because of the high energy density they can provide. However, the life of Li-Ion batteries is strongly conditioned by chemical- and mechanical-degradation mechanisms such as the formation of the so-called solid–electrolyte interface (SEI) or because of mechanical damage due to diffusion-induced stress (DIS) in active material particles [14,15]. Therefore, there is an urgent need to better understand these mechanisms that threaten the life of the batteries [16,17]. The role of diffusion-induced fracture in active particles of electrodes has increasingly been named as one of the critical factors limiting the capacity of the batteries [18,19,20,21,22]. Namely, cracks nucleate in the active particles when the material strength is surpassed due to DIS. Subsequent crack propagation through active particles leads to a loss in ion diffusivity and to an increase in SEI formation. Hence, the amount of lithium that can intercalate/deintercalate during further charge/discharge cycles of the battery is reduced. Determination of mechanical stress levels within the active particle is thus fundamental for estimating the evolution of mechanical degradation.

Several works have already studied the mechanical stresses and fracture phenomena of active particles in lithium-ion batteries. In one of the first studies of mechanical damage, Christensen and Newman [18] determined the distribution of mechanical stresses in carbonaceous battery anode materials. They analyzed the effect of volumetric expansions and the resulting hydrostatic stresses occurring during the intercalation of lithium ions in spherically-shaped active particles. Zhang et al. [23] described DIS in active particles resorting to a thermal analogy and assuming both perfect spherical and ellipsoidal shapes. Cheng and Verbrugge [24] derived analytical solutions of the mechanical stresses within active spherically-shaped particles under both galvanostatic and potentiostatic conditions. Continuum-based models using a phase-field approach have been successfully implemented for studying electrochemical phenomena [25,26,27]. Mukherjee and coworkers [28,29,30] introduced the use of a two-dimensional lattice spring-based model to describe microcrack nucleation and the subsequent propagation of microcracks in active graphite particles. Their model obtained the evolution of the lithium-ion concentration during lithiation and delithiation processes and the consequent mechanical degradation due to DIS.

To date, most models used to study the mechanical degradation in active particles consider simple shapes (spherical, ellipsoidal, or a combination of both). However, real particles present a variety of convex and concave surfaces. These surfaces can act as stress concentrators, thus increasing cracking risks during lithium-ion intercalation/deintercalation processes in the active particle [31]. In this work, we overcome the shape limitations present in current numerical descriptions of mechanical damage. We do so by introducing a novel random active particle generator. In particular, we generate active particles as three-dimensional n-faced convex polyhedrons.

Heterogeneity, disorder, and preexisting defects in graphite active particles can also play an important role in fracture processes. These effects have also been overlooked by the existing numerical models of the mechanical degradation of Li-ion batteries. Here, we account for these phenomena within the so-called lattice model framework [32,33,34]. In lattice models, the continuum (i.e., active particle) is discretized into a set of one-dimensional elements that host the diffusive and mechanical interactions of the material. Originally developed to describe fracture mechanisms in brittle disordered materials, such as graphite [35], lattice methods are perfectly suited for the numerical implementation of material heterogeneity and defects. In particular, we account for these here using a Weibull distribution function to describe mechanical properties (such as local diffusivity, elastic modulus, or strength) at the microscale. An additional benefit of the method is that it can spontaneously nucleate microcracks; whereas in previous models, cracks had to be preallocated.

Using our developed numerical framework we analyze the mechanical and functional degradation process in active graphite particles in lithium-ion batteries. We describe the effects of diameter, surface roughness, particle shape, and charging rate on complex fracture phenomena promoted by DIS. Namely, we provide details of crack nucleation, growth, and even branching or bending. Moreover, the presence of mechanical damage (cracks) limits ion-diffusion paths, leading to a reduced effective ion-diffusivity and uneven Li ion concentration gradients. In turn, these gradients further promote stress concentrations. This coupled phenomenon is conveniently captured by our model. In fact, we can identify the cycling behavior of the particles when subjected to different charging (and discharging rates). A direct consequence of impaired diffusion is a capacity loss of the battery, since the total amount of Li that can be intercalated is reduced. We use this idea to provide a qualitative estimate of battery capacity loss under different scenarios.

Aside from heterogeneities/imperfections, other localized effects such as abnormal local overheating are known to compromise battery performance [36], whose mechanism is similar to that of supercapacitors or pitting corrosion [37]. Nonhomogeneous temperature fields could be readily incorporated into our numerical model by inclusion of a heat diffusion equation. However, thepresent work is limited to a constant temperature field, and temperature effects will be part of future work. The present implementation of the method also assumes classical Fickian diffusion laws, as well as constant Li-ion flux throughout the active particle boundary. These assumptions become questionable at large C-rates; however, the qualitative behavior and trends displayed by our model provide useful information.

Despite the continuous effort in the search for alternative components, graphite is still the most used material in lithium-ion batteries due to its cycling stability and its high energy density. Thus, our results solely relate to graphite active particles, which make lithium-ion battery anodes. An active area of research towards improving electrodes focuses on the use of two-dimensional nanomaterials such as chalcogenides [38], MXenes [39], or rGO [40]. At the material level, the benefits of these novel electrodes can be qualitatively explored in our model using a reduced disorder parameter *s*, as well as a narrower Weibull distribution. At the component level, the performance of different nanostructures (e.g., layers and fibers) could be tested.

The numerical details of the implementation of the novel features introduced in our model are first described in Section 2 below. Our findings on the effects of the particle shape, material disorder, and charging rates on the fracture characteristics and the corresponding estimates of diffusivity and capacity losses are next summarized in Section 3.

## 2. Materials and Methods

We used a set of coupled initial and boundary value equations to describe the fracture processes due to diffusion-induced stresses in the active particles of the electrodes. Although our present work focused on the material level (that is, on active particles), the model can be readily extended to describe the behavior at the component level (i.e., the electrodes) and other materials such as silicon [41,42,43]. Our physical models, described below, were numerically solved using a lattice model formulation, that is, using one-dimensional elements within a finite element method framework.

We established the following model to capture the onset and propagation of damage within AP. Initially, the concentration of lithium ion within the particle was numerically obtained from the solution of a diffusion equation (Equation (3)). The transport of Li ions within graphite was modeled as a single-phase diffusion process. Then, mechanical stresses within the particle due to the contraction/expansion caused by the de-/lithiation process were evaluated. As a central assumption, mechanical damage was used to explain the degradation behavior of the materials due to microdamage and was coupled with diffusive damage.

### 2.1. Spatial Discretization

**Figure 1:**
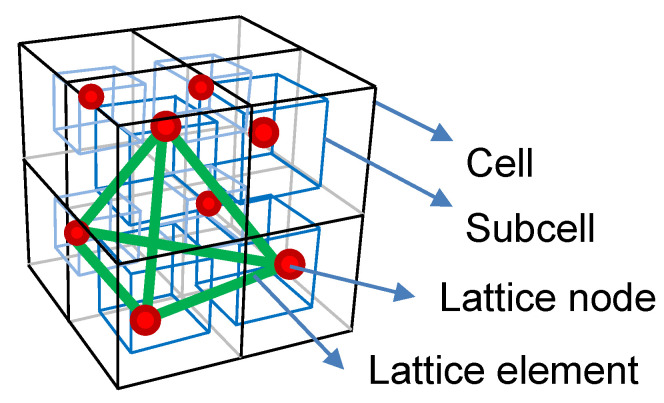
Node placement procedure and Delaunay tessellation.

The numerical framework used was the so-called lattice model: a discrete modeling technique that presents an alternative to continuous models for fracture analysis of brittle and disordered materials. In this approach, the material structure is discretized into a network of one-dimensional elements in which different physical phenomena take place. This results in a three-dimensional lattice. The nodes of this lattice were placed according to the following procedure. First, the domain was divided into cubic cells; then, a sub-cell was defined inside each cell. Nodes were randomly placed within these sub-cells (one node per sub-cell). The degree of randomness of the mesh, typically referred to as parameter *s*, was determined by the ratio between the size of the sub-cell and the cell. Higher values of *s* lead to more disordered meshes, while lower values of *s* lead to regular structures. Finally, a Delaunay tessellation was determined through the nodes, resulting in the lattice network (Figure 1). The mesh elements need to be assigned a cross-section value [44], which are calibrated comparing the numerical solution with the analytical results of representative problems (i.e., expansion or contraction of a sphere under external pressure, in our case).

### 2.2. Particle Generation

**Figure 2:**
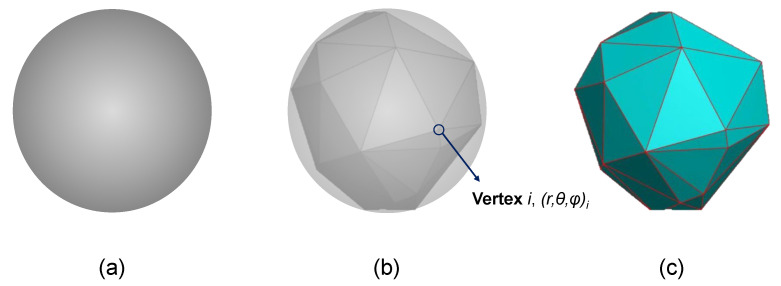
Construction of a convex polyhedron with n=30 vertices: (**a**) sphere as construction basis, (**b**) allocation of vertices on the sphere, and (**c**) the resulting polyhedron.

We developed a numerical algorithm to generate random geometries that mimic real nonspherical active particles. All generated particles are convex polyhedrons with tunable characteristics (e.g., number of vertices or size). The algorithm starts from an sphere. Then, a certain number of vertices are randomly placed on the surface. These points are next triangulated using Delaunay triangulation, yielding a convex polyhedron. The geometry obtained this way is thus characterized by the number of vertices chosen, as shown in Figure 2.

Vertices are randomly chosen according to the following method [45]:

(1) $$\left\{\begin{array}{c} x_{i}=a\sin\varphi_{i}\cos\theta_{i}\\ y_{i}=b\sin\varphi_{i}\sin\theta_{i}\\ z_{i}=c\cos\varphi_{i} \end{array}\right.$$

where the azimuthal and polar angles are given by:

(2) $$\left\{\begin{array}{c} \begin{array}{cc} \theta_{i}=\frac{2\pi }{n}+\delta \frac{2\pi }{n}\left(2\eta -1\right) & \forall i=1,\ldots ,n \end{array}\\ \begin{array}{cc} \varphi_{i}=2\pi \eta & \forall i=1,\ldots ,n \end{array} \end{array}\right.$$

where δ is a random number between 0 and 1, and 0 < η < 1.

**Figure 3:**
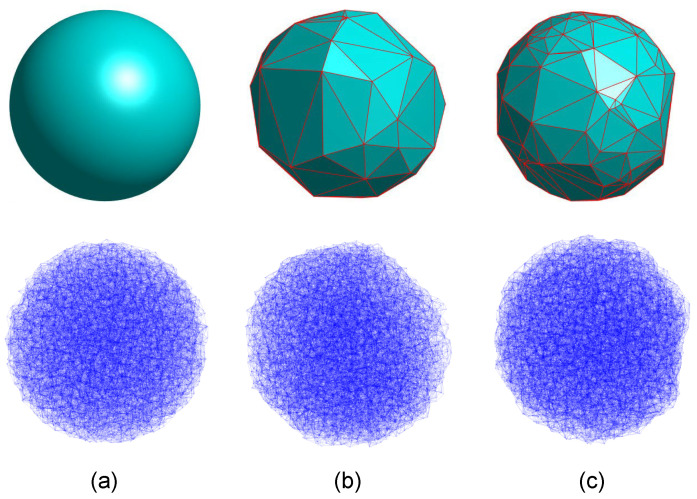
Lattice model meshing (bottom) and corresponding geometry (top): (**a**) sphere, (**b**) polyhedron n=60 vertices, and (**c**) polyhedron n=120 vertices.

Figure 3 shows two convex polyhedra generated, using 60 and 120 vertices (Figure 3b and Figure 3c, respectively), compared to the equivalent spherical particle (Figure 3a). The radius of the sphere from which the polyhedron is generated is adjusted depending on the type of problem to be studied. On the one hand, it can be increased so that the volume of the polyhedron obtained is equal to that of a perfect sphere (Figure 3a). In this way, the lithium-ion hosting capacity of the active particle is not reduced due to generating the polyhedron shape. On other hand, it can be kept as the original size of the particle, if we want to keep the maximum size.

### 2.3. Coupled Diffusive-Mechanical Model

The transport of Lithium ions in the active material is described using the following diffusion equation:

(3) $$\frac{\partial c}{\partial t}+\nabla \cdot J=0$$

where *c* is the concentration of Li ions in the active material, and J is their flux, which is given by:

(4) $$J=-D\left(\nabla c-\frac{\Omega c}{RT}\nabla \sigma_{h}\right)$$

where D is the Li diffusion matrix in the active material, *R* is the gas constant, Ω is the Li-ion partial molar volume, *T* is the absolute temperature (in this work, it is assumed to be constant), $\sigma_{h}$ is the hydrostatic stress (defined as $\sigma_{h}=\mathrm{tr}\left(\sigma \right)/3$), and σ is the stress tensor. Notice that, as is immediately apparent in Equation (4), Li transport is coupled to the mechanical problem, since the thermodynamic driving force for Li diffusion includes gradients in both the Li concentration and in the hydrostatic stress. We take $c\left(x,0\right)=c_{0}$ as the initial Li concentration in active particles.

The boundary conditions at the surface of the active particle follow from charge conservation considerations. That is, at the particle surface:

(5) $$J\cdot n=-\frac{i_{n}}{F}$$

where *F* is Faraday’s constant, and $i_{n}$ is the current density on the active particle surface (considered positive during the intercalation process and negative during the deintercalation process).

In this work, we assume that the ionic conductivity of the electrolyte is very large and neglect the ion concentration gradients in the electrolyte. Under these galvanostatic conditions, active particles are subjected to a constant current, and the boundary conditions simplify to:

(6) $$\frac{i_{n}}{F}=\mathrm{const}.$$

We define Li flux in terms of the C-rate (that is, the theoretical time required in hours to fully charge or discharge the battery) as follows:

(7) $$∥J∥=\frac{V}{3600S}c_{max}C$$

where *V* is the volume of the particle, *S* is the surface area of the particle, $c_{max}$ is the maximum stoichiometric ion concentration, and *C* is the C-rate.

The diffusion of Li ions, together with a nonuniform concentration field within the active particle, induces significant volumetric expansions/contractions. The particle deformation, in turn, causes internal stresses. The total strain field in the active particle, ε, can be related to the displacement field u within the domain by:

(8) $$\varepsilon =\frac{1}{2}\left(\nabla u+\nabla u^{⊤}\right).$$

The total strain tensor accounts for both the diffusive and mechanical strains in our coupled diffusive-mechanical model. The mechanical strains correspond to the deformation induced by the mechanical forces, while the diffusive strains correspond to the volume expansion (contraction) due to the lithiation (delithiation) stages imposed on the particle. That is,

(9) $$\varepsilon =\varepsilon^{el}+\varepsilon^{in}+\varepsilon^{d}$$

where $\varepsilon^{in}$ and $\varepsilon^{el}$ are, respectively, the inelastic and elastic components of the mechanical strain tensor, and $\varepsilon^{d}$ is the diffusion-induced strain field. Assuming an isotropic expansion, the latter can be defined as:

(10) $$\varepsilon^{d}=\frac{\Omega }{3}\left(c-c_{0}\right)I$$

where *c* is the concentration of Li ions in the active material, and $c_{0}$ is the initial lithium ion concentration in the active material at the stress-free state.

We use the continuum damage formulation of [46] for the constitutive law of the material, which reads as:

(11) $$\sigma =\left(1-d\right)D_{0}^{el}:\varepsilon^{el}$$

where $D_{0}^{el}$ is the initial (undamaged) elasticity matrix. In the formulation, the elastic stiffness degradation is characterized by the degradation parameter, *d*. The degradation parameter can take values ranging from zero (undamaged material) to one (corresponding to total loss of strength). In the description of the fracture of brittle materials (such as the case of graphite), $\varepsilon^{in}=0$, and the damage variable can take only two discrete values, i.e., d=0 or d=1.

Finally, we apply the equilibrium equation:

(12) ∇·σ+b=0

where σ is the stress tensor, and b the body force vector.

### 2.4. Lattice Model Implementation

In the lattice approximation of the diffusion problem, lithium ions’ transport within the active particle was modeled through a network of one-dimensional flow elements. On the other hand, the beam elements with shear correction were used for the mechanical problem. Since the diffusion of the ions takes place within the solid medium, the same lattice elements were used for both the mechanical and diffusive problems. However, the cross section of the elements for each problem were defined separately. In the case of the diffusion problem, the area of adjacent Voronoi facets was considered, as proposed by [47].

The diffusive-mechanical problem is time-dependent. For this reason, we need to establish a time-integration scheme, apart from the spatial FE-based discretization. The numerical implementation of the lattice model is summarized as follows. For each time step, the diffusive problem was solved in the first place. Then, once the Li-ion concentration in the active particle was determined, the diffusive forces were applied to the lattice elements, and the mechanical problem was solved. The element stresses were evaluated and checked with respect to the failure criterion. The elements that surpassed the failure surface became damaged elements, and the mechanical and diffusion matrices were updated.

The time-dependent transport problem in matrix form reads:

(13) $$M_{d}\dot{c}+K_{d}c=q$$

where q is the flux vector, and $M_{d}$ and $K_{d}$ are the mass and diffusion element matrices defined as:

(14) $$K_{d}=\frac{DA}{L}\left[\begin{array}{cc} 1 & -1\\ -1 & 1 \end{array}\right]$$

(15) $$M_{d}=\frac{AL}{6\omega }\left[\begin{array}{cc} 2 & 1\\ 1 & 2 \end{array}\right]$$

where *D* is the diffusion coefficient, *A* is the cross-sectional area of the element of the lattice, *L* is the length of the element, and ω is a correction parameter that accounts for the overlapping volume of adjacent lattice elements (ω=3 for three-dimensional lattice models [47]).

The concentration $c_{j}$ at time step $t_{j}$ can be expressed using the generalized central difference scheme [48]:

(16) $$c_{j}={\left(\frac{1}{\Delta t}M_{d}+\nu K_{d}\right)}^{-1}\left(\left(\frac{1}{\Delta t}M_{d}-\left(1-\nu \right)K_{d}\right)c_{j-1}+q_{j-1}\right)$$

where Δt is the time step. When ν=0.5, this integration scheme becomes the so-called Crank-Nicolson.

The mechanical problem at time step $t_{j}$ reads:

(17) $$K_{m}u_{j}=f_{j}$$

where $K_{m}$ is the global stiffness matrix, and $f_{j}$ is the global force vector at instant $t_{j}$ and includes the mechanical ($f_{m,j}$) and diffusion forces ($f_{d,j}$).

The global element matrix, $K_{m,\mathrm{eg}}$, is assembled using the corresponding transformation matrices with the direction cosines of the elements and the local stiffness element, which in the three-dimensional case is a 12×12 matrix as follows:

(18) $$K_{m,\mathrm{el}}=\left[\begin{array}{cccccccccccc} \frac{EA}{L} & 0 & 0 & 0 & 0 & 0 & -\frac{EA}{L} & 0 & 0 & 0 & 0 & 0\\ & \frac{12EI}{L^{3}\left(1+\mu \right)} & 0 & 0 & 0 & \frac{6EI}{L^{2}\left(1+\mu \right)} & 0 & -\frac{12EI}{L^{3}\left(1+\mu \right)} & 0 & 0 & 0 & \frac{6EI}{L^{2}\left(1+\mu \right)}\\ & & \frac{12EI}{L^{3}\left(1+\mu \right)} & 0 & -\frac{6EI}{L^{2}\left(1+\mu \right)} & 0 & 0 & 0 & -\frac{12EI}{L^{3}\left(1+\mu \right)} & 0 & -\frac{6EI}{L^{2}\left(1+\mu \right)} & 0\\ & & & \frac{GJ}{L} & 0 & 0 & 0 & 0 & 0 & -\frac{GJ}{L} & 0 & 0\\ & & & & \frac{(4+\mu )EI}{L(1+\mu )} & 0 & 0 & 0 & \frac{6EI}{L^{2}\left(1+\mu \right)} & 0 & \frac{(2-\mu )EI}{L(1+\mu )} & 0\\ & & & & & \frac{(4+\mu )EI}{L(1+\mu )} & 0 & -\frac{6EI}{L^{2}\left(1+\mu \right)} & 0 & 0 & 0 & \frac{(2-\mu )EI}{L(1+\mu )}\\ & & & & & & \frac{EA}{L} & 0 & 0 & 0 & 0 & 0\\ & & & & & & & \frac{12EI}{L^{3}\left(1+\mu \right)} & 0 & 0 & 0 & -\frac{6EI}{L^{2}\left(1+\mu \right)}\\ & & & & & & & & \frac{12EI}{L^{3}\left(1+\mu \right)} & 0 & \frac{6EI}{L^{2}\left(1+\mu \right)} & 0\\ & & & & & & & & & \frac{GJ}{L} & 0 & 0\\ & & & & & & & & & & \frac{(4+\mu )EI}{L(1+\mu )} & 0\\ sym. & & & & & & & & & & & \frac{(4+\mu )EI}{L(1+\mu )} \end{array}\right]$$

where *E* is the elastic modulus, *A* is the cross-section, *L* is the length of the element, *I* is the inertia, *G* is the shear modulus, *J* is the polar moment of inertia about the *x*-axis, and μ is the shear-correction factor, which in the case of a circular cross section becomes μ=10/9.

In every mechanical step, the elements that surpassed the failure surface were removed (i.e., their damage variable was set to 1) generating microcracks. To determine the equivalent stress of the element, we considered only the axial interaction: $\sigma_{eq}\;=\;\frac{N}{A}$, where *N* is the axial force in the element. Thus, when the equivalent stress of the element surpassed the material tensile strength, $\sigma_{eq}>f_{t}$, the element underwent failure.

Finally, the diffusive and mechanical element matrices were updated according the damage variable of the elements.

## 3. Results and Discussion

In this work, we analyzed the degradation mechanisms in graphite active particles of Li-ion battery anodes. For this purpose, we followed a numerical approach combining the particle generator along with the coupled diffusive-mechanical model presented in Section 2. We took into account the cycling phenomena by driving a galvanostatic lithiation process at different charging rates (i.e., 0.5C, 1C, and 2C) until stoichiometric conditions, followed by its corresponding delithiation process. Moreover, we considered different particle shapes and the internal material disordered. The material properties considered in the simulations are shown in Table 1.

**Figure 4:**
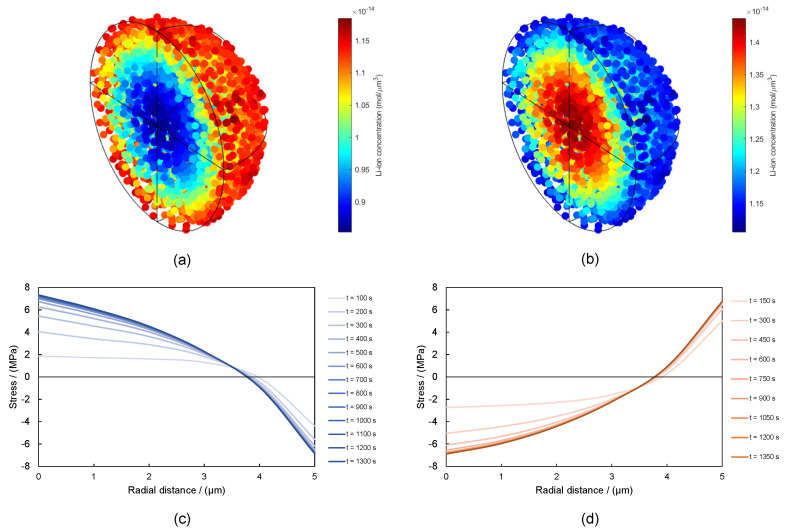
Li-ion concentration: (**a**) lithiation (t = 200 s) and (**b**) delithiation (t = 200 s). Stress evolution during (**c**) lithiation and (**d**) delithiation.

During lithiation, ions flowed from the outer surface of the particle towards the core, until the maximum Li-ion concentration in graphite was reached (denoted by $c_{max}$). This generated a concentration gradient toward the core of the particle that induced the outer region of the particle to swell, generating compressive hoop stresses close to the surface. To satisfy the geometric compatibility conditions, tensile stresses developed at the center (Figure 4a). For this reason, cracking initiated from the core during lithiation, since brittle materials (such as the graphite) are much more prone to fracture under tensile stresses [50]. On the other hand, during delithiation, the mechanism was the opposite: as Li-ions departed from the particle, compressive stresses developed in the core, and tensile stresses appeared on the outer surface (Figure 4b). As we observe in Figure 4, this was a time-dependent phenomenon, and the most important stress gradients took place during the first seconds of the process. This crack formation mechanism has been discussed in models of the cracking behavior of active Li-ion batteries particles, both in early and current mathematical models [14,18], as well as in numerical–experimental models [51,52,53] and also in 3D tomography of single particles at different states of charge [54]. For the sake of clarity, we have not included the failure process in these results (Figure 4). These are accounted for hereafter.

### 3.1. Effect of Particle Shape

One of the main features of our modeling framework is its ability to account for different types of particle shapes. While most of the simulation works assume perfect spherical shapes for the active particles [55], it is well known that these typically present irregular shapes [56]. With our particle generator, based on the construction of polyhedra, we are able to realize particles with irregular shapes. Thus, using a spherical particle as a reference with diameter 10 μm [57], we established two comparisons: (i) polyhedra with the same maximum size and (ii) polyhedra with the same volume. In the first case, we generated polyhedra with a different number of vertices placed on a wrapping sphere of same diameter as the reference spherical particle. In this case, the volume enclosed by the polyhedron was smaller than that of the sphere. In the second case, we kept the volume of the polyhedra constant with respect to that of the sphere by reallocating the vertices and, thus, increased the maximum size of the particle. In such a way, we yielded an equivalent particle in terms of storage capacity.

**Figure 5:**
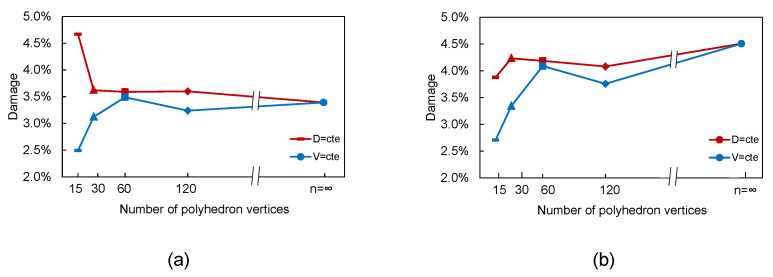
Damage level in the polyhedra as a function of the number of the vertices following the constant particle size and volume approaches: (**a**) lithiation at 1C and (**b**) delithiation at 1C.

In Figure 5, we present the damage level of the particle at 1C, which was measured as the cumulative element damage, i.e., the addition of the contribution of each broken element to the damage. In the case of brittle failure, the element damage was defined as the ratio of the cross section of the broken element to the sum of all the cross sections. Therefore, the damage level of the particle can be measured as the sum of the cross sections of the elements that failed over the sum of the cross sections of all the elements. The results are presented for the two cases considered: (i) keeping the maximum particle size (denoted as D=cte) and (ii) keeping the particle volume (denoted as V=cte). A wide range of polyhedron vertices were considered, namely *n* = 15, 30, 60, and 120. We also included a case with a large number of vertices (n>500), which we refer to as n→∞. Furthermore, since fracture evolution during lithation differs from that of delithiation, we present both cases.

In the case of lithation (Figure 5a), we can observe that as we increased the number of the vertices of the polyhedron, the damage level yielded the same value as expected (when n→∞, both approaches yielded the shape of a spherical particle). However, it can be seen that, for low values of vertices, more irregular shapes were accomplished; thus, the damage results varied. If we follow a constant volume approach, the total damage was underestimated, while in the constant size approach, the total damage was overestimated. Thus, the assumption of a perfectly spherical particle can yield relative errors of up to 40% when evaluating the cumulative damage instead of using an irregular shape with same maximum size. This difference was less, i.e., 28%, when the irregular particle had the same volume.

These differences were also observed in the case of delithiation (Figure 5b). However, in this case, we reached up to 60% when we considered equivalent volumes, while this number was reduced to 13% when the equivalent size was kept. This is mainly due to the fact that failure driven by delithiation initially takes place in the outer surface, while in lithiation it starts in the core of the particle.

**Figure 6:**
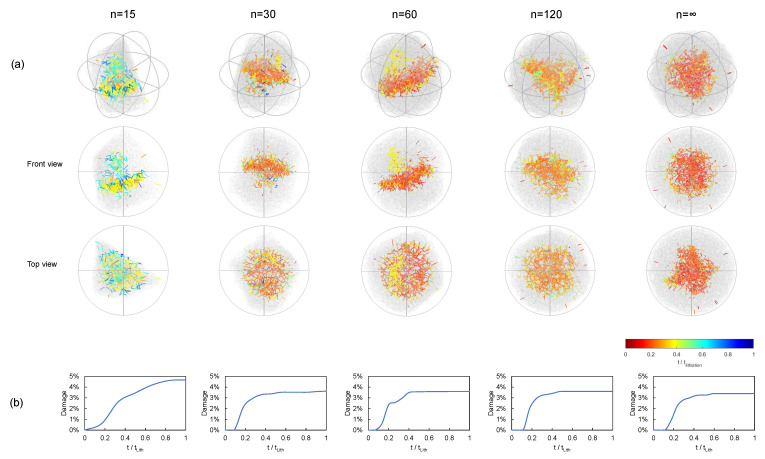
Damage evolution in terms of the particle shape. Pure lithiation, C-rate = 2, polyhedra of constant size: (**a**) Crack pattern evolution and (**b**) Damage evolution.

**Figure 7:**
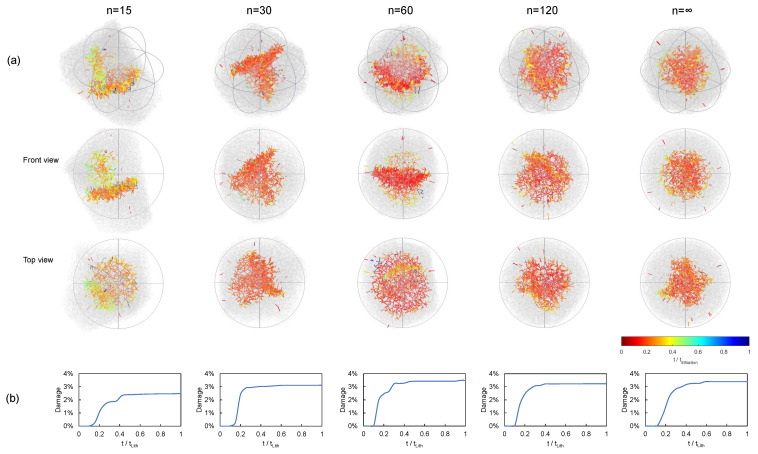
Damage evolution in terms of the particle shape. Pure lithiation, C-rate = 2, polyhedra of constant volume: (**a**) crack pattern evolution and (**b**) damage evolution.

In Figure 6, we present the cracking patterns of different particle shapes during lithiation and considering the maximum particle size as constant. These are shown for polyhedra of vertices *n* = 15, 30, 60, 120, and *∞*. The color map represents the evolution of microcracks from the first instants of the lithiation process (in red) until the maximum concentration was reached (in blue). In Figure 6b, we represent the corresponding cumulative damage evolution. From these results, it can be observed that, to a large extent, the majority of the fracture process occurred within 0.2 and 0.4$t_{lith}$, where $t_{lith}$ is the time required to achieve the maximum stoichiometric concentration. The cracks were initially nucleated at the core of the particles and propagated towards the surface in different fracture planes. These were influenced by the shape of the particle and, as expected, the cumulative damage became larger for smaller values of *n*. In general terms, we observe a cumulative damage of approximately 4%. On the other hand, when the particle volume was kept (Figure 7), we observe similar cracking patterns, but the resulting damage was lower.

**Figure 8:**
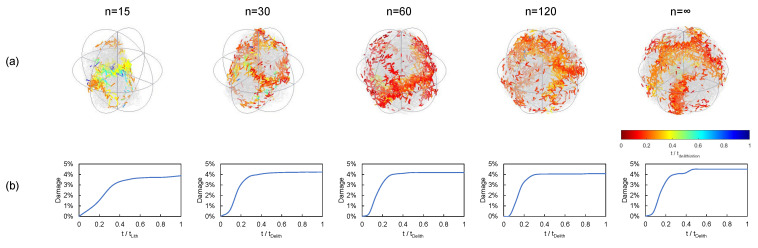
Damage evolution in terms of the particle shape. Pure delithiation, C-rate = 2, polyhedra of constant size: (**a**) crack pattern evolution and (**b**) damage evolution.

**Figure 9:**
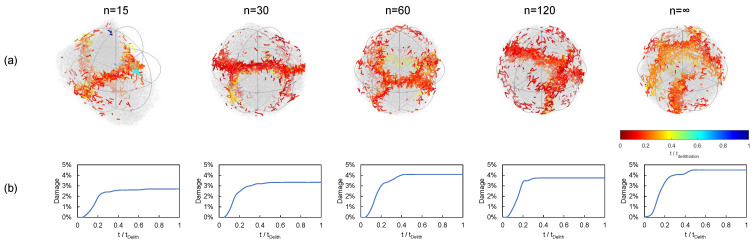
Damage evolution in terms of the particle shape. Pure delithiation, C-rate = 2, polyhedra of constant volume: (**a**) crack pattern evolution and (**b**) damage evolution.

As pointed out above, delithiation promotes the apparition of tensile stresses close to the outer surface due to the migration of ions leaving the particle. This results in surface cracks that propagate towards the core. This is observed in Figure 8 and Figure 9. In general terms, the cumulative damage yielded levels of 4%, and most of the fracture events occurred within the first 0.4$t_{delith}$ time window, where $t_{delith}$ is the time required to completely delithiate the particle. It must be remarked that our model was able to represent complex fracture behavior including crack nucleation and growth, branching, and change in direction, resembling actual cracking patterns observed in the literature [58].

### 3.2. Effect of Material Disorder

Graphite presents a highly disordered internal material structure [59,60], and this is typically neglected in most of the numerical models of graphite particles. Although our numerical model partially accounted for this through the so-called randomness ratio *s*, we also made use of probability distribution functions to incorporate additional variability in the local material properties. Namely, we used Weibull’s probability density function [61,62] to account for the imperfections in graphite particles. We applied this function to the element elastic modulus so as to intrinsically account for potential defects, such that:

(19) $$E_{i}\;=E_{0}{\left[\ln\frac{1}{{1-\omega }_{i}}\;\right]}^{1/m}$$

where $E_{i}$ is the elastic modulus of the i-th element, $E_{0}$ is the global elastic modulus of graphite, $\omega_{i}$ is a random number ranging from 0 to 1, and *m* is the Weibull’s modulus or shape parameter. In our simulations, m=3. A greater value of *m* implies more homogeneity of the model.

In the first place, we analyzed the effect of material disorder in a perfectly spherical particle subjected to a full lithiation–delithiation cycle at different C-rates. In Table 2, we can observe that accounting for this phenomenon resulted in important differences in the damage level. For instance, at 1C, the obtained cumulative damage with perfect material properties was 2.6%. This value increased up to 5.5%, i.e., more than twice the original value. As the C-rate increased, the damage level increased, and this trend was also observed in our simulations, obtaining larger damage values for Weibull’s model (35.8%) versus the homogeneous model (24.2%).

**Figure 10:**
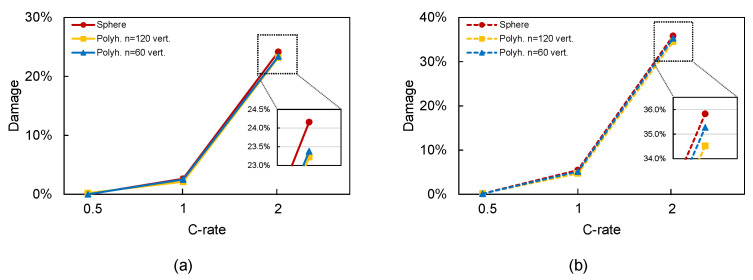
Effect of particle shape on damage after 1 cycle: (**a**) homogeneous material properties and (**b**) non-homogeneous material properties.

We also analyzed the effect of particle shape along with the material disorder (see Figure 10). For the sake of objectivity, we considered more regular particle shapes (i.e., n=60 and 120. The damage level achieved with homogeneous properties (Figure 10a) was lower than that of the Weibull’s model (Figure 10b): 23–24% versus 35–36% for 2C. Of course, adding material inhomogeneity leads to more spread cracking patterns; thus, the damage level is more affected. In any case, it can be observed that differences in the damage level reached 122% at 1C and 51% in the case of 2C, which are relevant.

**Figure 11:**
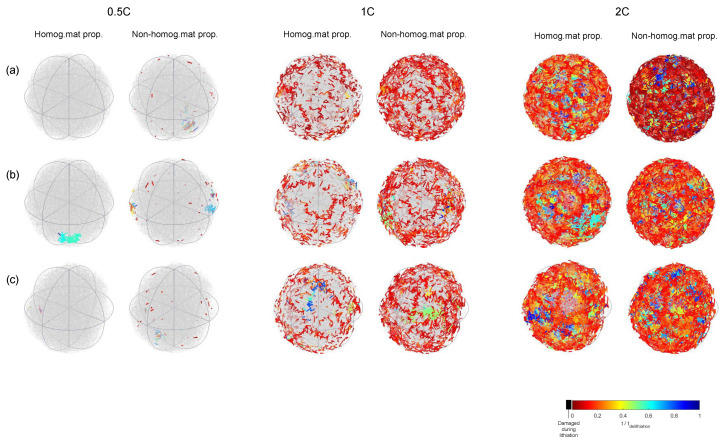
Damage distribution after 1 cycle: (**a**) sphere, (**b**) polyhedron (n=120), and (**c**) polyhedron (n=60). Homogeneous and heterogeneous material properties.

Figure 11 shows the cracking pattern evolution within one lithiation–delithiation cycle for different particle shapes and C-rates. In the first place, it can be observed that, as the C-rate increased, cracking density increased. It can also be observed that the nonhomogeneous cases (i.e., Weibull’s model) presented a higher cracking density than from the homogeneous model.

### 3.3. Diffusivity and Capacity Loss

Lithium ion transport takes place within the active material, which in our case is graphite. As discussed above, the lithiation and delithiation processes result in cracking patterns that depend on different variables (e.g., C-rate, material heterogeneity, and particle shape, to name a few). From a mechanical point of view, microcracks can be seen as internal material zones without any bearing capacity (i.e., they cannot withstand stresses). Thus, cracks become paths through which Li ions can no longer migrate or be stored. From the transport phenomena point of view, this promotes a loss of storage capacity in the particle.

Since cracking is both a spatial and temporal process, we discuss here how this loss of capacity evolves, first, from the perspective of the effective diffusivity of lithium in the particle matrix and, second, with a simple estimate of the total amount of Li that can be stored in the active particle.

**Figure 12:**
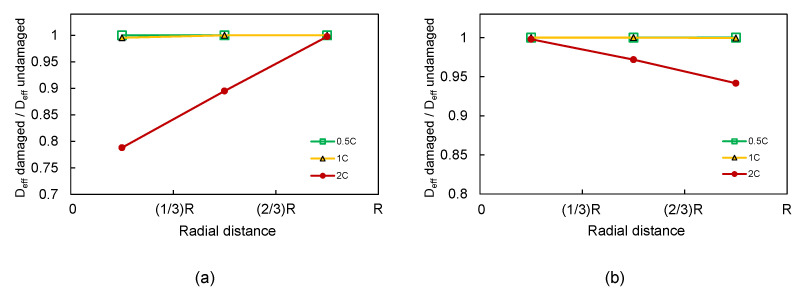
Loss of diffusivity at different radial distances for different C-rates: (**a**) pure lithiation and (**b**) pure delithiation. Spherical particle with heterogeneous material properties.

Figure 12 shows the reduction in orientation- and volume-averaged effective diffusivity for a spherical particle of 10 μm, as a function of radial position. The effective diffusivity at a given radial position was obtained by averaging the diffusivity over all polar and azimuthal angles in a differential spherical shell. During lithiation (Figure 12a), we observe that the reduction in diffusivity of the particle increased as we moved closer to the core. This can be easily explained in terms of cracking patterns: cracks nucleate near the core and grow outwards, leaving the outer surface hardly affected. We can also observe that the diffusivity reduction was strongly dependent on the C-rate, with diffusivity losses up to 20% near the core for 2C. As expected, during delithiation (Figure 12b), the radial dependence of the diffusivity reduction was reversed. The results in this figure correspond to heterogeneous material properties (i.e., Weibull’s model).

**Figure 13:**
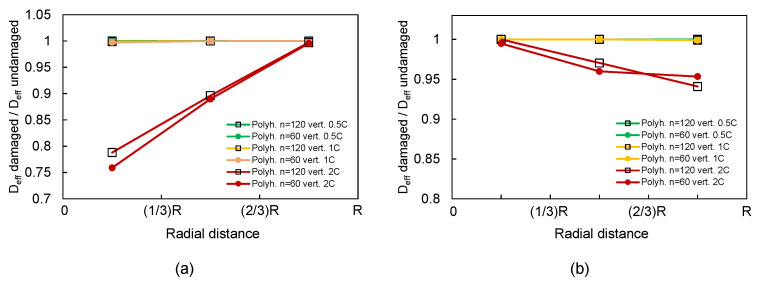
Loss of diffusivity at different radial distances for different C-rates: (**a**) pure lithiation and (**b**) pure delithiation. Polyhedra particles (n=60 and 120) with heterogeneous material properties.

Figure 13 presents the diffusivity loss as a function of the radial distance for different particle shapes (n=60, and 120) and different C-rates. The maximum diffusivity loss increased to 25% in the case of n=60 and 2C, which was the most extreme case considered in lithiation (see Figure 13a). While the effect was milder during delithiation (Figure 13b), the reduction was yet greater, more than 5% than for the spherical case.

The reduction in the orientation-averaged effective diffusivity bears a direct relation to the loss of the storage capacity of the battery. However, a more precise relation between mechanical damage and battery performance degradation needs to be established. A very simple estimate of capacity loss can be obtained from the maximum amount of Li that can be intercalated, at a given charging rate, during a single lithiation step, before the maximum concentration of Li is reached at the particle surface. Since, in our simulations, we imposed a constant Li flux at the boundary, we can simply obtain an estimate of capacity loss with respect to an undamaged state as the ratio between total charging time (for given charging rate) of the damaged state and total charging time of the reference (pristine) particle state. While an admittedly overly simplified model of battery degradation, capacity loss defined this way after one cycle is shown in Table 3 for different C-rates. As expected, the higher the C-rate, the higher the capacity loss. We also find that this relationship was not linear, and while in the case of 1C the capacity loss was around 5% in the first cycle, it surpassed 10% for 2C.

## 4. Conclusions

Lithium-ion batteries currently find widespread use as storage technologies in many different applications such as consumer electronics, electric vehicles, or renewable energies. Their main advantage is the high energy density of the active materials in the anode, namely, graphite. On the other hand, these batteries suffer from capacity loss with cycling, particularly at large charging rates. Diffusion-induced fracture is one important mechanism that leads to a loss of the capacity in the active material. In this work, we detailed a novel modeling framework to gain insight into the complex coupled phenomena, namely, ion transport and material damage. The numerical results were discussed to provide, for different scenarios, (i) insight into the mechanisms of mechanical damage (i.e., details on crack nucleation and evolution) and (ii) an estimate of battery capacity loss.

Specifically, we discussed a novel 3D formulation of the so-called lattice model applied to graphite active particles. Our numerical model solved a strongly coupled species transport–mechanical problem. In the transport problem, gradients in hydrostatic stress were one of the driving forces for Li-ion diffusion. Additionally, Li-ion diffusivity in the active particle was made dependent on mechanical damage through a damage parameter that modeled reduced diffusion paths as cracks appeared in the material. On the other hand, mechanical stresses in graphite (and consequently crack nucleation and propagation) depended on volumetric changes induced by Li-ion concentration in the active particle.

Our numerical model introduced important novelties over previous works. It included fracture capabilities; i.e., it can predict crack nucleation, whereas previous models assume an elastic regime and preallocate cracks. Moreover, the effect of material disorder and/or preexisting defects can be readily accounted for in the model. In addition, particles of arbitrary shape can be easily studied. Thus, we also developed an algorithm to numerically generate n-face polyhedra that mimicked actual active particles. In contrast to previous studies that assume simple geometries (e.g., disks, ellipses, spheres, and ellipsoids), our algorithms can account for stress concentration due to realistic irregular shapes.

The numerical results of the pure lithiation and delithiation analyses showed that particle shape had an important effect on global damage. Specifically, damage in irregularly-shaped particles can be up to 60% larger than on equivalent spherical particles. Increased damage is mainly the result of stress concentration that only occurs for non-smooth irregular shapes.

We also discussed cracking pattern evolution both in lithiation and delithiation processes. Our results showed that most of the failure events in the material occurred within the first half of the process ($0.4t_{lith}$ and $0.4t_{delith}$). We also found that the maximum damage (obtained for C-rate 2) remained below 5%. Cracking patterns were displayed for different charging conditions (0.5C, 1C, and 2C), particle shapes (e.g., vertices, maximum size, and volume), and at different times. The results showed good qualitative agreement with the experimental observations. Moreover, we are able to reproduce complex fracture patterns such as crack nucleation, growth, branching, change of direction, etc.

The details of the internal material structure also played an important role in fracture mechanisms. We accounted for particle microstructure in two ways: introducing a tunable randomness parameter in the mesh and using a Weibull’s probability distribution function to specify the physical properties of the material. In this way, both the internal disorder present in graphite and implicit imperfections were included in our model. In our results, we found differences in particle damage depending on microstructure that can be up to 110%. This shows that the internal microstructure plays a fundamental role in particle degradation and subsequent capacity loss.

Finally, we analyzed the effect of lithiation/delithiation on a position-dependent effective Li-ion diffusivity and on the capacity loss of active particles. As cracks are barriers to Li-ion diffusion, mechanical damage resulted in a reduced effective diffusivity. We found local effective diffusivity was reduced up to 25% close to the core of the particle due to lithiation, and reduced in ca. 5% close to the outer surface due to delithiation (for C-rate 2). Reduced diffusivity leads to larger Li-ion concentration gradients. Therefore, for a given charging rate, the total amount of Li (and thus, battery capacity) that can be intercalated in the active particle before a maximum concentration is reached is reduced with mechanical degradation. Using a simple estimate based on this idea, we found that the C-rate had a nonlinear effect on battery degradation: particle cycling led to 5% capacity loss at 1C and was larger than 10% at 2C.

In future work, we will explore the effect of other material features such as the orientation of the basal planes of graphite or the role of internal defects in degradation, along with the formation of the solid–electrolyte interface, which are of great importance in degradation mechanisms.

## Figures and Tables

**Table 1:** Material properties for graphite.

Material Property (Units)	Graphite [49]
*E* (GPa)	15
ν	0.3
$f_{t}$ (MPa)	35
*D* (m^2^/s)	7.08 × 10^−15^
Ω (m^3^/mol)	1.14 × 10^−6^
$c_{max}$ (mol/m^3^)	22,900

**Table 2:** Effect of material disorder on damage after 1 cycle (spherical-shaped particle).

C-Rate	Damage	
	Homogeneous Model	Heterogeneous Model
0.5C	0.00%	0.18%
1C	2.64%	5.46%
2C	24.16%	35.83%

**Table 3:** Capacity loss for different particle shapes and C-rates.

C-Rate	Capacity Loss		
	Sphere	Polyhedron (n=120)	Polyhedron (n=60)
0.5C	3.14%	3.11%	2.41%
1C	5.79%	4.73%	5.32%
2C	11.56%	12.44%	10.66%

## Data Availability

Not applicable.
